# Selections from the French

**Published:** 1888-03

**Authors:** 


### SELECTIONS FROM THE FRENCH.

TRANSLATED BY F. J. ARBEELY, M. D., ATLANTA, GA.

THE ESSENCE OF TURPENTINE AS AN INJECTION IN FISTULA.—Dr. Cecchini uses the essence of turpentine in various forms of fistula with the greatest success. According to his view this was due to the irritant, modifying, healing and a specially antiseptic action of turpentine; he declares its superiority even to the best disinfectants known, as carbolic acid, salicylic acid, naphtol and the bichloride of mercury; he uses it pure with better and more rapid action, or mixed with olive oil or sweet almond oil to moderate the severe pain sometimes produced from its use. In a timid or nervous patient he injects a solution of muriate of morphine first, or he makes a mixture of the two, as there is no counter-action between them; by this means he reduces the pain to a minimum; in all cases he uses Pravaz syringe. The results of Dr. Cecchini's experiments are these:

Out of 7 cases of anal fistula 5 were cured, treatment discontinued in one and a negative result from the other case; six cases of caries in the vertebra were cured; eight cases of dental fistula, complicated with caries extending more or less to the maxillary bone, were treated by the same method with very happy results, except in one case in which the fistula was in Steno's duct; besides, fifteen more cases of various forms of fistula which met with the same result under this treatment.—(*Annali Univers di Medicina.*)

UNCONTROLLABLE VOMITING IN PREGNANCY CURED BY ALIMENTATION WITH A TUBE IN THE ŒSOPHAGUS.—Brünniche gives an account of a woman who was irregular in her monthly period; for two months she suffered from a grave gastric trouble, was admitted to the hospital, where an ulcer of the stomach was the diagnosis without the possibility of pregnancy. Shortly after her admission the vomiting became so grave that the patient rejected all kinds of nourishment; even life was threatened with inanition; alimentation with an œsophageal tube was practiced; an injection of broth, followed by another of cold water, was given; both injections were retained; on another occasion milk then broth were introduced in the same manner with the same result, in five days nourishment was given without the aid of a tube; the nausea and vomiting returned; then resort to the tube was necessary, but for three weeks more only, when the pregnancy was assured and the woman cured and delivered afterward.

We observe in this case that the sensitive region which caused the vomiting when irritated is located above the stomach and higher up in the digestive channel, for all that is necessary to do is to introduce the tube to the upper part of the œsophagus only.—(*Centralblatt fur Gynœkologie.*)

AGARICINE IN THE SWEATING OF PHTHISIS.—Piermy, the author, has experimented recently in Professor Pribrams clinic with this new alkaloid, prepared from the alcoholic extract of the white Agaric; he gives it in doses from [illegible]/70 to [illegible] of a grain without any effect sometimes, but generally it diminishes a profuse sweating when he gives it in $\frac{3}{70}$ of grain or more; he had the following results:

(1.) Agaricine nearly always decreases the sweating in consumptives.

(2.) It has no modifying effect on the respiration.

(3.) In cases of profuse sweating, when suppressed by this alkaloid, the pulmonary and skin functions were not modified.

(4.) The result seems to depend on lessening the absorption of water as the thirst and urine were diminished under its use.

(5.) A few pills of $\frac{1}{6}$ of a grain each of agaricine would arrest a profuse sweating in four or five hours.

(6.) The use of agaricine gives no inconvenience whatever afterwards.

(7.) The feebleness of the consumptive is diminished, but the other symptoms are not modified.—(*Farmacesta Italiano et Jour. de Med. et Farmacol.*

